# Is Pain Intensity Related to Psychosocial Factors in Chronic Non‐Nociceptive Orofacial Pain Patients?

**DOI:** 10.1111/joor.70094

**Published:** 2025-11-07

**Authors:** Akiko Kawase, Hitoshi Higuchi, Fumika Hashimoto, Saki Miyake, Yukiko Nishioka, Midori Inoue, Hitomi Ujita, Aki Kawauchi, Shigeru Maeda, Takuya Miyawaki

**Affiliations:** ^1^ Department of Dental Anesthesiology and Special Care Dentistry Okayama University Graduate School of Medicine, Dentistry and Pharmaceutical Sciences Okayama Japan; ^2^ Department of Dentistry Ohara Health Care Foundation Kurashiki Central Hospital Kurashiki Japan; ^3^ Department of Dental Anesthesiology Okayama University Hospital Okayama Japan; ^4^ Department of Dental Anesthesiology Graduate School of Medical and Dental Sciences, Institute of Science Tokyo Tokyo Japan

**Keywords:** chronic pain, International Classification of Orofacial Pain, orofacial pain, psychological distress component, psychosocial factors, self‐efficacy/QOL component

## Abstract

**Background:**

In order to understand the psychological aspects of chronic pain, it is important to consider the relationships between pain and psychosocial factors in patients with chronic pain. While psychosocial factors are known to affect pain intensity in temporomandibular disorders, few studies have evaluated them in patients with other types of chronic orofacial pain.

**Objective:**

The purpose of the present study was to evaluate the relationships between pain intensity and patient characteristics, diagnostic categories and psychosocial factors in chronic non‐nociceptive orofacial pain patients.

**Methods:**

In a retrospective, cross‐sectional study, we collected information from the medical records of 123 patients with chronic non‐nociceptive orofacial pain. Pain intensity was measured using the Brief Pain Inventory (BPI) total score. Analysis of the correlations among the variables revealed several strong correlations. Principal component analysis identified two components: the psychological distress and self‐efficacy/quality of life (QOL) components. Multiple linear regression analyses of the overall study population and each ICOP pain category were also performed.

**Results:**

In the overall sample, higher BPI scores were significantly associated with a greater psychological distress component and lower self‐efficacy/QOL component. The pain category was not a significant predictor of the BPI score. In the subgroup analyses, both components were significant predictors of the BPI score in myofascial orofacial pain; whereas, only the self‐efficacy/QOL component was in idiopathic orofacial pain.

**Conclusion:**

The results indicated that pain intensity in chronic non‐nociceptive orofacial pain is related to the self‐efficacy/QOL psychosocial factor component. These findings suggest that assessing psychosocial factors may be clinically important for the diagnosis and treatment of chronic orofacial pain.

## Graphical abstract

Chronic non‐nociceptive orofacial pain is affected by psychosocial factors related to the self‐efficacy/QOL component, which was composed of the Pain Self‐Efficacy Questionnaire (PSEQ) and EuroQoL 5‐Dimensions (EQ‐5D).

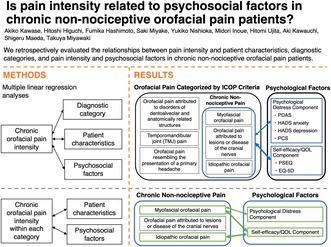

## 1 Introduction

Orofacial pain is defined as a form of frequent pain experienced in the face and/or oral cavity [1]. In the orofacial region, the most frequently observed type of pain, nociceptive pain, is thought to be triggered by the acute stimulation of nociceptors by various oral stimuli [2]. Nociceptive pain initially arises as acute pain, but is self‐limiting and restricted to a specific period of time [3]. However, when pain persists beyond the healing of the injured tissue and the resolution of the associated inflammatory processes, it is considered chronic. Chronic pain is defined as pain that persists or recurs for more than 3 months [4]. Chronic pain is considered to be affected by certain sociopsychological conditions and vice versa [5]. A large‐scale study by Macfarlane et al. [6] reported a prevalence of facial pain in the UK of 1.9%, of which 48% was chronic, suggesting that orofacial pain becomes chronic at a relatively high rate.

In order to understand the psychological aspects of chronic pain and to treat them more appropriately, it is important to consider patients' psychosocial factors. Many studies on the relationships between chronic pain and psychosocial factors have been reported [7, 8, 9], and there have also been many studies of orofacial pain, but most of these examined patients with temporomandibular joint disorder (TMD) [10, 11, 12]. However, few studies have evaluated these relationships in patients with other types of chronic orofacial pain. Furthermore, we considered that the relationships between pain intensity and psychosocial factors differ between chronic nociceptive pain and other types of chronic pain.

At our facility, all patients with orofacial pain undergo an assessment of pain intensity using the Brief Pain Inventory. Additionally, the following instruments are used to assess various psychosocial factors, including pain‐related life disorders, at the initial visit as part of patients' medical treatment: the Hospital Anxiety and Depression Scale, Pain Catastrophizing Scale, Pain Self‐Efficacy Questionnaire, Pain Disability Assessment Scale, EuroQoL 5‐Dimensions and Athens Insomnia Scale. Consequently, a substantial body of data concerning pain intensity and psychosocial factors, including pain‐related life disorders, in patients with orofacial pain has been amassed.

Therefore, the purpose of the present study was to evaluate the relationships between pain intensity and diagnostic category, the examined patient characteristics, and psychosocial factors in chronic non‐nociceptive orofacial pain patients. The International Classification of Orofacial Pain (ICOP) criteria [13] were used as diagnostic categories. Orofacial pain attributed to disorders of dentoalveolar and anatomically related structures, myofascial orofacial pain and TMJ pain, as categorised by the ICOP criteria, were classified as nociceptive pain [14]. However, it has been suggested that myofascial pain may develop nociplastic features when it becomes chronic [15, 16]. Therefore, patients with myofascial orofacial pain, orofacial pain attributed to lesions or disease of the cranial nerves, and idiopathic orofacial pain were included as subjects in the present study.

## 2 Methods

### 2.1 Subjects

This was a retrospective observational cross‐sectional study. We studied patients who visited our facility complaining of orofacial pain between January 2019 and December 2023. All patients consented to the use of their individual anonymized data for research purposes. The patients' diagnoses were determined using the diagnostic criteria developed by the ICOP [13], which are as follows:

**Table:** 

Category 1:	Orofacial pain attributed to disorders of dentoalveolar and anatomically related structures Explanation: Pain caused by disease, injury, or abnormal functioning of the tooth pulp, periodontium, gingiva(e), oral mucosa, salivary glands or jawbone tissue or pain arising from normal functioning of the tooth pulp signalling risk of tooth damage
Category 2:	Myofascial orofacial pain Explanation: Pain localised to the masticatory muscles, with or without functional impairment
Category 3:	Temporomandibular joint (TMJ) pain Explanation: Pain localised to the TMJ, occurring at rest or during jaw movement or palpation
Category 4:	Orofacial pain attributed to lesions or disease of the cranial nerves Explanation: Pain localised in the distribution area of one of the sensory cranial nerves (i.e., the trigeminal or glossopharyngeal nerve) with a history of trauma or disease known to cause nerve injury
Category 5:	Orofacial pain resembling the presentation of a primary headache Explanation: Pain in the orofacial area, resembling one of the types of primary headache in pain character, duration and intensity with or without the associated symptoms of this headache type, but without a concomitant headache
Category 6:	Idiopathic orofacial pain Explanation: Unilateral or bilateral intraoral or facial pain in the distribution(s) of one or more branches of the trigeminal nerve(s) for which the aetiology is unknown
Category 7:	Psychosocial assessment of patients with orofacial pain Explanation: Not applicable

The examination and diagnosis of patients were performed by dentists specialising in orofacial pain at our hospital's pain center. As the ICOP was published in 2020, for patients evaluated prior to its publication, the ICOP criteria codes were retrospectively applied to their diagnoses, based on information obtained from their medical records.

The subjects of the present study were screened using the following criteria:

#### 2.1.1 Inclusion Criteria

- 20 years old or older
- Pain in the orofacial region lasting more than 3 months
- Cooperated fully and was able to complete all of the questionnaires used to assess pain intensity and psychosocial factors, including pain‐related life disorders

#### 2.1.2 Exclusion Criteria

- Nociceptive pain
- Major diseases, such as cancer, affecting the oral or maxillofacial region
- Major surgery within 6 months
- Critical mental disorders, such as bipolar disorder or depression

### 2.2 Outcomes

#### 2.2.1 Primary Outcomes

The primary outcomes were to evaluate the relationships between pain intensity and patient background factors (sex, age and duration of pain), diagnostic categories and psychosocial factors in patients with orofacial pain.

#### 2.2.2 Secondary Outcomes

The secondary outcomes were to evaluate the relationships between pain intensity and patient background factors (sex, age and duration of pain) and psychosocial factors within each diagnostic category.

### 2.3 Measures

Age, sex, the duration of the current pain, and the diagnosis were obtained from the patients' medical records as patient characteristics. Data regarding pain intensity and psychosocial factors, including pain‐related life disorders, were obtained from the questionnaire completed by each patient at their initial visit. The questionnaire was a self‐administered, multiple‐choice questionnaire composed of the following seven items:

#### 2.3.1 Brief Pain Inventory (BPI)

On a scale of 0 (no pain) to 10 (worst pain imaginable), the patients were asked to indicate the most appropriate pain level for each of the following four items: the most intense pain level experienced in the past 24 h, the weakest pain level experienced in the past 24 h, the average pain level experienced in the past 24 h, and their current pain level [17]. The total score for these four items was used as the BPI total score.

#### 2.3.2 Pain Disability Assessment Scale (PDAS)

The PDAS assesses the degree to which pain interferes with various activities of daily living and is a tool for assessing pain‐related life disability. It is commonly used to evaluate the characteristics of pain without specifically limiting the area affected by the pain [18]. It consists of 20 items, which are assessed on a 4‐point scale, from 0 (no difficulty in performing activity) to 3 (unable to perform activity because it is too painful) for each item. The total score ranges from 0 to 60, with higher scores indicating more severe pain‐related life disorders.

#### 2.3.3 Hospital Anxiety and Depression Scale (HADS)

The HADS assesses patients' psychological state regarding anxiety and depression [19]. The questionnaire consists of seven items related to anxiety and seven items related to depression. Each item is rated on a scale of 0 to 3, resulting in a maximum total score of 21 for both anxiety and depression, with higher scores indicating greater severity.

#### 2.3.4 Pain Catastrophizing Scale (PCS)

The PCS assesses the extent to which patients catastrophize about their pain [20]. The questionnaire consists of 13 items, each of which includes three subscales: rumination, magnification and helplessness. Each item is rated on a 5‐point scale ranging from 0 (not at all) to 4 (all the time). Scoring involves summing all of the item scores and calculating a total score, which ranges from 0 to 52. The higher the score, the more catastrophic the patient views their pain.

#### 2.3.5 Pain Self‐Efficacy Questionnaire (PSEQ)

The PSEQ assesses how confident a person is that they can do various activities at present, despite the pain they are experiencing [21]. The questionnaire consists of 10 items, each of which includes three subscales. The three subscales are each rated on a 7‐point scale, ranging from 0 (not at all confident) to 6 (completely confident). Scoring involves summing all of the item scores to generate a total score, which ranges from 0 to 60, with higher scores indicating higher levels of self‐efficacy.

#### 2.3.6 EuroQoL 5‐Dimensions (EQ‐5D)

The EQ‐5D health utility score is computed by converting the responses to the five items of the EQ‐5D questionnaire (i.e., questions regarding mobility, self‐care, usual activities, pain and discomfort and anxiety and depression) to one summary value. This calculation is done with country‐specific value sets. A value set for Japan is available [22].

#### 2.3.7 Athens Insomnia Scale (AIS)

The AIS assesses the degree of insomnia using diagnostic criteria established by the International Classification of Diseases (ICD‐10) [23]. The eight‐item questionnaire assesses sleep onset, nighttime and early morning awakening, sleep duration, sleep quality, the frequency and duration of complaints, distress caused by experiencing insomnia and interference with daily functioning. Scoring involves summing all of the item scores and calculating a total score, which ranges from 0 to 24. The higher the score, the more severe the insomnia.

### 2.4 Data Analysis

Statistical analyses were performed for the patient characteristics (age, sex, duration of pain and ICOP criteria) and for pain intensity and the PDAS, HADS (anxiety), HADS (depression), PCS, PSEQ, EQ‐5D and AIS scores. Spearman's rank correlation analysis (two‐tailed) was used to determine the correlations between the variables. Multicollinearity and singularity are potential problems that can affect a correlation matrix when variables are too strongly correlated. We first summarised the subjects' characteristics and examined bivariate relationships using Spearman's rank correlation coefficients (two‐tailed). As several psychosocial variables showed moderate‐to‐high intercorrelations (|*r*| > 0.5, with two pairs > 0.7), we evaluated the suitability of dimension reduction using the Kaiser–Meyer–Olkin (KMO) measure and Bartlett's test of sphericity. Both indices supported factorability; therefore, we conducted principal component analysis (PCA) of seven psychosocial measures (the HADS‐A, HADS‐D, AIS, PCS, PDAS, PSEQ and EQ‐5D). Two components were identified, the psychological distress component (comprising the HADS‐A, HADS‐D, AIS, PCS and PDAS) and the self‐efficacy/QOL component (comprising the PSEQ and EQ‐5D). Component scores were computed (using the regression method) for the subsequent modelling.

Multiple linear regression was then performed, with the BPI total score used as the dependent variable. The primary model for the overall study population included age, sex, pain duration, the psychological distress component, the self‐efficacy/QOL component, and the ICOP pain category (dummy‐coded with myofascial orofacial pain as the reference). To explore diagnosis‐specific patterns, separate models were estimated within each ICOP category (myofascial, idiopathic and cranial‐nerve lesion pain). Multicollinearity was assessed using tolerance and the variance inflation factor (VIF); all VIF values were < 2, indicating no multicollinearity. Missing data were handled by listwise deletion. All analyses were conducted in SPSS version 20, and *p*‐values < 0.05 were considered significant.

### 2.5 Ethics

Ethical approval for the present study was granted by the institutional ethics committee (K1907‐041).

## 3 Results

### 3.1 Subjects

**FIGURE 1:**
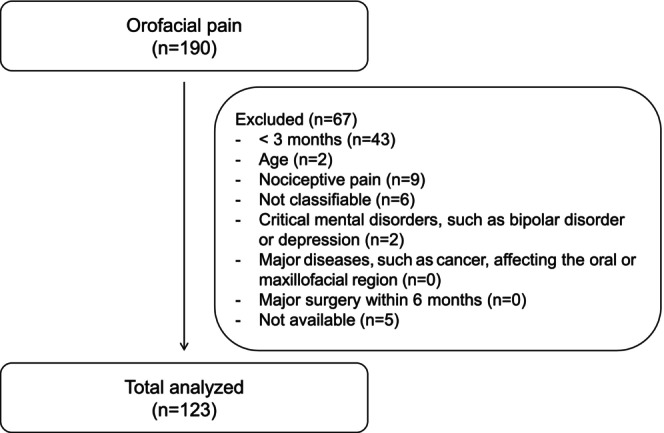
Participant recruitment process.

There were 190 patients who visited our facility complaining of orofacial pain between January 2019 and December 2023. Of them, 67 patients were excluded from the present study because they were less than 20 years old; had nociceptive and/or inflammatory pain, such as temporomandibular joint pain; or pain lasting less than 3 months. Therefore, the number of subjects included in the present study was 123 (Figure 1). The characteristics of the subjects are shown in Table 1. The subjects ranged in age from 20 to 90 years (mean age: 56.2 ± 16.3 years) and consisted of 31 males and 92 females. The median duration of their pain was 12 months. The numbers and percentages of subjects in each ICOP criteria are shown in Table 2. Idiopathic orofacial pain was the most common type of pain (51 subjects, 41.4%), followed by myofascial orofacial pain (44 subjects, 35.8%). None of the subjects had orofacial pain caused by disorders of dentoalveolar or anatomically related structures or temporomandibular joint pain because such patients were excluded from the present study. None of the patients had presentations resembling primary headaches.

**TABLE 1:** Descriptive statistics for patient characteristics, pain intensity and various clinical assessments.

Patient characteristics		
Age, years, mean (SD)	56.2 (16.3)	
Sex, *n* (%) (female/male)	92 (74.8%)/31 (25.2%)	
	**Median (quartiles)**	**Mean (SD)**
Duration of current complaints, months	12.0 (6.0–28.0)	30.5 (47.5)
Pain intensity		
Highest BPI in the last 24 h	5.0 (3.0–8.0)	5.4 (2.5)
Lowest BPI in the last 24 h	2.0 (1.0–3.0)	2.3 (2.2)
Mean BPI	4.0 (3.0–6.0)	4.4 (2.3)
Current BPI	3.5 (2.0–6.0)	4.1 (2.5)
BPI total score	14.5 (10.0–22.0)	16.2 (8.6)
Clinical assessments		
PDAS	16.0 (5.0–24.0)	16.7 (12.9)
HADS anxiety	6.0 (3.5–8.0)	6.2 (4.1)
HADS depression	5.5 (3.0–9.0)	6.2 (4.5)
PCS	30.5 (24.0–38.0)	30.4 (10.2)
PSEQ	33.0 (22.0–43.0)	32.1 (15.0)
EQ‐5D	0.7 (0.6–0.8)	0.6 (0.2)
AIS	6.0 (3.5–11.0)	7.8 (5.2)

Abbreviations: AIS, Athens Insomnia Scale; BPI, Brief Pain Inventory; EQ‐5D, EuroQol 5‐Dimensions; HADS, Hospital Anxiety and Depression Scale; PCS, Pain Catastrophizing Scale; PDAS, Pain Disability Assessment Scale; PSEQ, Pain Self‐Efficacy Questionnaire.

**TABLE 2:** Diagnostic categories based on the ICOP criteria.

		Study sample (*n* = 123)	Percent
1	Orofacial pain attributed to disorders of dentoalveolar and anatomically related structures	0	0.0
2	Myofascial orofacial pain	44	35.8
3	Temporomandibular joint (TMJ) pain	0	0.0
4	Orofacial pain attributed to lesions or disease of the cranial nerves	28	22.8
5	Orofacial pain resembling the presentation of a primary headache	0	0.0
6	Idiopathic orofacial pain	51	41.4

Abbreviation: ICOP, the International Classification of Orofacial Pain.

### 3.2 Relationships Between Pain Intensity and Other Variables

#### 3.2.1 Primary Outcomes

The results of the correlation analysis are shown in Table 3. Pain intensity, as measured by the BPI total score, was positively correlated with the PDAS (*r* = 0.570, *p* < 0.0001), HADS (depression) (*r* = 0.269, *p* = 0.037), and PCS (*r* = 0.450, *p* < 0.0001) scores and negatively correlated with the PSEQ (*r* = −0.450, *p* < 0.0001) and EQ‐5D (*r* = −0.586, *p* < 0.0001) scores. However, pain intensity was not related to age, the duration of pain or the HADS (anxiety) or AIS score.

**TABLE 3:** Correlations between variables.

	BPI total score	Age	Duration of pain	PDAS	HADS‐A	HADS‐D	PCS	PSEQ	EQ‐5D	AIS
BPI total score		0.130	−0.079	0.570**	0.118	0.269*	0.450**	−0.450**	−0.586**	0.208
Age		—	0.044	0.147	−0.102	−0.087	−0.009	−0.003	0.016	−0.036
Duration of pain			—	0.016	0.176	0.170	−0.039	−0.089	0.016	0.000
PDAS				—	0.308*	0.419**	0.303*	−0.574**	−0.644**	0.314*
HADS‐A					—	0.733**	0.307*	−0.382**	−0.446**	0.500**
HADS‐D						—	0.336**	−0.592**	−0.612**	0.542**
PCS							—	−0.403**	−0.400**	0.186
PSEQ								—	0.758**	−0.485**
EQ‐5D									—	−0.457**
AIS										—

*Note:* *p < 0.05, **p < 0.01.

Abbreviations: AIS, Athens Insomnia Scale; BPI, Brief Pain Inventory; EQ‐5D, EuroQol 5‐Dimensions; HADS, Hospital Anxiety and Depression Scale; PCS, Pain Catastrophizing Scale; PDAS, Pain Disability Assessment Scale; PSEQ, Pain Self‐Efficacy Questionnaire.

The results of the multiple regression analysis of the overall study population are shown in Table 4. In the regression model in which the BPI total score was used as the dependent variable (*R* ^2^ = 0.483), both the psychological distress component (B = 1.99, 95% confidence interval [CI]: 0.04–3.94, *p* = 0.046) and the self‐efficacy/QOL component (B = 5.29, 95% CI: 3.24–7.35, *p* < 0.001) were identified as significant predictors of the BPI total score, whereas the ICOP pain category was not.

**TABLE 4:** Multiple linear regression of the associations between the BPI total score and various clinical assessments.

Variables	B	95% CI	*p*
Constant	13.724	10.645–16.802	< 0.001
Psychological distress component	1.988	0.036–3.939	0.046
Self‐efficacy/QOL component	5.292	3.237–7.346	< 0.001
Idiopathic orofacial pain (vs. myofascial orofacial pain)	2.977	−1.355–7.309	0.174
Orofacial pain attributed to lesions or disease of the cranial nerves (vs. myofascial orofacial pain)	4.616	−1.502–10.734	0.136

*Note:* Adjusted *R* ^2^ = 0.483. The psychological distress component was comprised of the HADS‐A, HADS‐D, AIS, PCS and PDAS. The self‐efficacy/QOL component was comprised of the PSEQ and EQ‐5D.

#### 3.2.2 Secondary Outcomes

To further examine diagnosis‐specific associations, separate models were analysed for each ICOP category (Table 5). In the myofascial orofacial pain group (*n* = 22), both the psychological distress component (B = 2.98, *p* = 0.028) and self‐efficacy/QOL component (B = 5.35, *p* = 0.001) were found to be significant predictors of the BPI total score. In the idiopathic orofacial pain group (*n* = 25), only the self‐efficacy/QOL component was shown to be a significant predictor of the BPI total score (B = 5.97, *p* < 0.001). In the cranial‐nerve lesion group (*n* = 7), neither component reached statistical significance.

**TABLE 5:** Multivariate linear regression analysis of predictors of pain intensity in each diagnostic category.

Variables	B	95% CI	*p*
A. Myofascial orofacial pain (*n* = 22)			
Constant	13.487	10.373–16.602	< 0.001
Psychological distress component	2.984	0.364–5.604	0.028
Self‐efficacy/QOL component	5.347	2.459–8.235	0.001
B. Orofacial pain attributed to lesions or disease of the cranial nerves (*n* = 7)			
Constant	22.706	14.026–31.386	0.002
Psychological distress component	4.116	−5.559–13.792	0.303
Self‐efficacy/QOL component	−7.223	−21.304–6.857	0.227
C. Idiopathic orofacial pain (*n* = 25)			
Constant	16.858	13.933–19.784	< 0.001
Psychological distress component	0.302	−2.781–3.385	0.841
Self‐efficacy/QOL component	5.972	3.011–8.934	< 0.001

*Note:* The psychological distress component was comprised of the HADS‐A, HADS‐D, AIS, PCS and PDAS. The self‐efficacy/QOL component was comprised of the PSEQ and EQ‐5D.

All models satisfied the assumptions regarding the independence of residuals, and no evidence of multicollinearity (all VIF < 2) or undue influence from extreme cases was found.

## 4 Discussion

Painful TMD is the most commonly studied type of pain in the orofacial region [10, 11, 12]. However, TMJ pain, which is a type of TMD, was excluded from the present study. TMJ pain has been reported to be inflammatory in nature [24] and is classified as a form of nociceptive pain [14]. Inflammatory pain is described as being due to increased sensitivity of perceptual and emotional responses to harmful stimuli caused by tissue injury‐related inflammatory reactions, and it is categorised as one of the most representative subtypes of nociceptive pain [25]. On the other hand, Safieh‐Garabedian et al. [26] reported that chronic inflammation following a nerve injury may promote neuropathic pain. However, to the best of our knowledge, there have not been any reports that TMJ inflammation directly causes neuropathic pain. For the reasons given above, TMJ pain, which was distinguished from myofascial orofacial pain based on the ICOP criteria, was excluded from the present study.

On the other hand, chronic myofascial orofacial pain was included in the present study. Myofascial pain is generally considered to be caused by the activation of nociceptors, for example due to a muscle injury, hypertonicity, or inflammation, and thus, is regarded as a type of nociceptive pain. However, it has been suggested that in chronic cases, especially when the pain persists despite tissue healing and the absence of ongoing nociceptor activation, myofascial pain may involve central sensitization and be classified as nociplastic pain [27]. Consistent with this finding, it has been suggested that myofascial pain may develop nociplastic features when it becomes chronic [15, 16].

Therefore, our results may differ from previous studies that investigated painful TMD cases involving both TMJ pain and myofascial orofacial pain. In patients with TMD, the association between pain and catastrophizing was reported to be a predictor of poor prognosis. Furthermore, although pain intensity in TMD was reported to be significantly associated with sleep disturbance [28, 29], no strong relationship between pain intensity and sleep disturbance was found in our study. It seems reasonable that TMD is related to sleep disturbance because nocturnal bruxism is a major cause of TMD, but the finding that non‐nociceptive orofacial pain is not strongly related to sleep disturbance represents a significant difference from the pain caused by TMD.

The most prevalent type of chronic pain is lower back pain, which is a common issue in primary care. Lower back pain is a typical form of chronic pain, but it has been reported that most patients have non‐organic lower back pain with no specific nociceptive source identified [30], and such pain is considered to be non‐nociceptive and may lead to a decline in the ability to perform activities of daily living [7, 8, 9]. Therefore, research on chronic pain has been driven by research on lower back pain. Chronic lower back pain reduces patients' quality of life and causes adverse effects, such as insomnia, anxiety, depression and decreased physical activity, and these biopsychosocial effects have economic consequences and are treated as social problems [31]. A previous study indicated that patients with chronic lower back pain who have a reduced sense of life control, mood disturbances, negative pain self‐efficacy, high anxiety levels and/or mental health disorders, and/or who engage in catastrophizing tend to not respond well to treatment [32]. The tendency towards lower pain self‐efficacy is consistent with the findings we obtained in patients with chronic orofacial pain. The evaluation of psychosocial problems is useful for identifying patients with a poor prognosis. Questionnaires, such as the PDAS, HADS, PCS, PSEQ, EQ‐5D and AIS, are the tools most commonly used for assessing the psychosocial problems associated with back pain. These scales have been used extensively in the field of lower back pain. Since these scales have been validated with respect to lower back pain, we also used them for the present study.

The results of the present study showed that pain intensity in patients with chronic orofacial pain was strongly associated with the self‐efficacy/QOL component, which was composed of the PSEQ and EQ‐5D. Low PSEQ and EQ‐5D scores suggest that patients with stronger chronic orofacial pain have lower pain self‐efficacy, meaning less confidence in their ability to carry out daily activities, and a worse health‐related quality of life. The EQ‐5D was reported to be related to symptoms of chronic pain [33]. This can be interpreted as indicating that PSEQ and EQ‐5D scores are reduced by orofacial pain. In other words, this may in part reflect a loss of confidence in one's ability to carry out daily activities or an increased awareness of declining health as a consequence of orofacial pain. Furthermore, in the present study, the psychological distress component was also shown to be a significant, but weak, independent predictor of pain intensity. Since the psychological distress component was composed of anxiety (HADS‐A), depression (HADS‐D), insomnia (AIS), catastrophic thinking (PCS) and the impairment of activities of daily living (PDAS), the psychological distress component may be a predictor of orofacial pain, while changes in these variables may be brought about by orofacial pain itself and/or its symptoms, such as catastrophic thinking and sleep disturbance. However, as the results were obtained from a cross‐sectional study, causal inferences cannot be drawn. It remains plausible that pain intensity may influence these psychological and functional domains, or that bidirectional relationships exist between them.

On the other hand, psychosocial factors, such as socioeconomic status, traumatic life events, social support and an inability to work, have also been reported to influence pain intensity, particularly in chronic orofacial pain [34, 35]. These findings suggest that psychosocial factors may play a significant role in shaping pain experiences. Moreover, this has potential clinical relevance, as modifying psychosocial factors through psychological interventions or cognitive‐behavioural therapy may contribute to pain reduction, as demonstrated in previous studies [36, 37, 38, 39].

The present study had the following limitations. The first is that the target population was the outpatients of a simple facility. Therefore, the results may have been biased and may not be widely applicable; however, the obtained findings were consistent with previous findings regarding chronic pain affecting other body areas and may have clinically significant implications for the treatment of chronic orofacial pain. Second, the sample size was not sufficient for a more rigorous analysis. Due to sample size limitations, we were only able to perform a limited analysis of diagnostic categories based on the ICOP criteria. If the sample size had been bigger, we could have analysed the data by diagnostic category based on the ICOP criteria, age group, or other background factors. Third, in the present study, the patients' disease history and clinical courses, including the type and number of interventions undertaken before the initial consultation, were not analysed because reliable information on these factors could not be obtained for all participants. These could be important factors that may have influenced the results. Fourth, because this was a cross‐sectional study future research should employ longitudinal or interventional designs to clarify the causal pathways between psychosocial factors and pain intensity in individuals with chronic orofacial pain. In particular, randomised controlled trials evaluating the efficacy of cognitive‐behavioural therapy, mindfulness‐based interventions, or social support enhancement programs may provide valuable insights into the modifiability of these factors and their impact on pain outcomes. Additionally, incorporating multidimensional assessments, including psychological, functional and social domains, could facilitate a more comprehensive understanding of the mechanisms underlying chronic pain and inform the development of personalised treatment strategies.

## 5 Conclusion

The present study focused on chronic non‐nociceptive orofacial pain, using the ICOP criteria, and evaluated the relationships between pain intensity and diagnostic categories, the examined patient characteristics, and psychosocial factors. The results indicated that pain intensity in chronic non‐nociceptive orofacial pain is affected by psychosocial factors related to the self‐efficacy/QOL component, which was composed of the PSEQ and EQ‐5D. These findings suggest that assessing psychosocial factors, including pain‐related life disorders, may be clinically important for the diagnosis and treatment of chronic orofacial pain.

## Author Contributions

Akiko Kawase, Hitoshi Higuchi, Fumika Hashimoto, Yukiko Nishioka, Saki Miyake, Midori Inoue, Hitomi Ujita, Aki Kawauchi, Shigeru Maeda and Takuya Miyawaki had full access to all of the data used in the study and take responsibility for the integrity of the data and the accuracy of the data analysis. Concept and design: Akiko Kawase, Hitoshi Higuchi and Takuya Miyawaki. Acquisition of data: Akiko Kawase, Fumika Hashimoto, Yukiko Nishioka, Saki Miyake, Midori Inoue and Hitomi Ujita. Analysis and interpretation of data: Akiko Kawase, Fumika Hashimoto, Yukiko Nishioka, Saki Miyake, Midori Inoue and Hitomi Ujita. Additional data analysis: Aki Kawauchi and Shigeru Maeda. Drafting of the manuscript: Akiko Kawase and Hitoshi Higuchi. Critical revision of the manuscript for important intellectual content: Hitoshi Higuchi and Takuya Miyawaki. Obtained funding: Akiko Kawase. Supervision: Takuya Miyawaki.

## Conflicts of Interest

The authors declare no conflicts of interest.

## Data Availability

The datasets generated during and/or analysed during the current study are available from the corresponding author on reasonable request.
